# Impact of Body Mass Index on the relationship of epicardial adipose tissue to metabolic syndrome and coronary artery disease in an Asian population

**DOI:** 10.1186/1475-2840-9-29

**Published:** 2010-07-07

**Authors:** Jin-Sun Park, Sung-Gyun Ahn, Jung-Won Hwang, Hong-Seok Lim, Byoung-Joo Choi, So-Yeon Choi, Myeong-Ho Yoon, Gyo-Seung Hwang, Seung-Jea Tahk, Joon-Han Shin

**Affiliations:** 1 Department of Cardiology, Ajou University School of Medicine, Suwon, Korea; 2 Division of Cardiology, Yonsei University Wonju College of Medicine, Wonju, Korea

## Abstract

**Background:**

In a previous study, we demonstrated that the thickness of epicardial adipose tissue (EAT), measured by echocardiography, was increased in patients with metabolic syndrome (MS) and coronary artery disease (CAD). Several studies on obese patients, however, failed to demonstrate any relationship between EAT and CAD. We hypothesized that body mass index (BMI) affected the link between EAT and MS and CAD.

**Methods:**

We consecutively enrolled 643 patients (302 males, 341 females; 59 ± 11 years), who underwent echocardiography and coronary angiography. The EAT thickness was measured on the free wall of the right ventricle at the end of diastole. All patients were divided into two groups: high BMI group, ≥27 kg/m^2 ^(n = 165), and non-high BMI group, < 27 kg/m^2 ^(n = 478).

**Results:**

The median and mean EAT thickness of 643 patients were 3.0 mm and 3.1 ± 2.4 mm, respectively. In the non-high BMI group, the median EAT thickness was significantly increased in patients with MS compared to those without MS (3.5 vs. 1.9 mm, p < 0.001). In the high BMI group, however, there was no significant difference in the median EAT thickness between patients with and without MS (3.0 vs. 2.5 mm, p = 0.813). A receiver operating characteristic (ROC) curve analysis predicting MS revealed that the area under the curve (AUC) of the non-high BMI group was significantly larger than that of the high BMI group (0.659 vs. 0.506, p = 0.007). When compared to patients without CAD, patients with CAD in both the non-high and high BMI groups had a significantly higher median EAT thickness (3.5 vs. 1.5 mm, p < 0.001 and 4.0 vs. 2.5 mm, p = 0.001, respectively). However, an ROC curve analysis predicting CAD revealed that the AUC of the non-high BMI group tended to be larger than that of the high BMI group (0.735 vs. 0.657, p = 0.055).

**Conclusions:**

While EAT thickness was significantly increased in patients with MS and CAD, the power of EAT thickness to predict MS and CAD was stronger in patients with BMI < 27 kg/m^2^. These findings showed that the measurement of EAT thickness by echocardiography might be especially useful in an Asian population with a non-high BMI, less than 27 kg/m^2^.

## Background

Central obesity (visceral adipose tissue) correlates strongly with the development of metabolic syndrome (MS) and coronary artery disease (CAD) [1]. There are ways to estimate visceral adipose tissue (VAT). Recently, there are several reports that proposed echocardiographic epicardial adipose tissue (EAT) as an easy and reliable imaging indicator of VAT. It is widely known that EAT thickness measured by echocardiography has a good correlation with abdominal adipose tissue measured by computed tomography (CT) and magnetic resonance imaging (MRI) [2,3].

In our previous study, the EAT thickness increased linearly as the MS score increased and the EAT was thicker in patients with MS than in those without MS. The EAT was thicker in patients with significant CAD than in those without CAD. We suggested that EAT thickness measured by echocardiography was a good surrogate marker for MS and CAD [3,4]. In another study, EAT thickness was also related to MS and the presence and extent of CAD [5].

Several groups have failed to demonstrate the correlation between EAT and CAD. Chaowalit et al. reported that EAT thickness is not associated with the presence, extent or severity of CAD in patients [6]. Gorter et al. reported that neither pericardial fat nor visceral abdominal fat correlated with CAD in patients with body mass index (BMI) ≥25 kg/m^2 ^[7]. In these reports, the subjects were relatively obese compared with the subjects in our previous study. In our study, the mean BMI of the subjects was 25.2 ± 3.1 kg/m^2^. In the report of Chaowalit et al., the mean BMI of the subjects was 28.8 ± 5.5 kg/m^2 ^[6]. The inconsistent results regarding the correlation between EAT and CAD might be due to differences in BMI of the study population. In one study, the correlation between pericardial fat and coronary plaque was seen in the non-obese patients but not in the overweight patients [8]. To evaluate the correlation between pericardial fat and CAD, it has been suggested that it is necessary to divide study subjects according to the degree of obesity, as obesity itself is frequently associated with various metabolic and circulatory factors and is known to be an independent risk factor for CAD [9]. Mazur et at. failed to demonstrate the correlation between EAT and MS. In that report, the subjects included obese children with BMI > 97 percentile as defined by the International Obesity Task Force [10,11]. In 2004, Kip et al. reported that the clinical effects of VAT on patients with MS differed between the normal BMI group and the obese group. In that study, the patients with MS and normal BMI had poorer clinical outcome compared to the patients with MS who were obese [12]. We hypothesized that the predicting ability of EAT thickness for MS and CAD is affected by BMI.

## Methods

### Study population

The EAT thickness was measured consecutively in 643 patients (mean age 59 ± 11 years; 302 males, 341 females) who underwent their first coronary angiography due to chest pain. The medical records of all patients were retrospectively reviewed after informed consent was obtained from patients. EAT has been proposed as a source of several inflammatory mediators [13-15]. We tried to exclude the acute systemic inflammatory effect to avoid confounding the role of EAT and diagnosis of MS. We excluded patients from the study if they had any of the following: active inflammation, a history of prior revascularization, heart failure, cardiomyopathy or acute myocardial infarction. We identified MS if there were more than 3 of 5 criteria based on the updated Adult Treatment Panel III (ATP III) guidelines. The MS score was defined as the number of criteria present. Adjusting values specifically for an Asian population, central obesity was defined as waist circumference of ≥90 cm in men and ≥80 cm in women [16]. Upon quantitative analysis of the coronary angiograms, significant CAD was considered to be the presence of one or more stenoses, ≥50% in diameter, of a major epicardial vessel. The major epicardial vessels were defined as the left main coronary artery that divides into the left anterior descending and circumflex branches, and the right coronary artery.

### Measurement of echocardiographic epicardial adipose tissue

**Figure 1:**
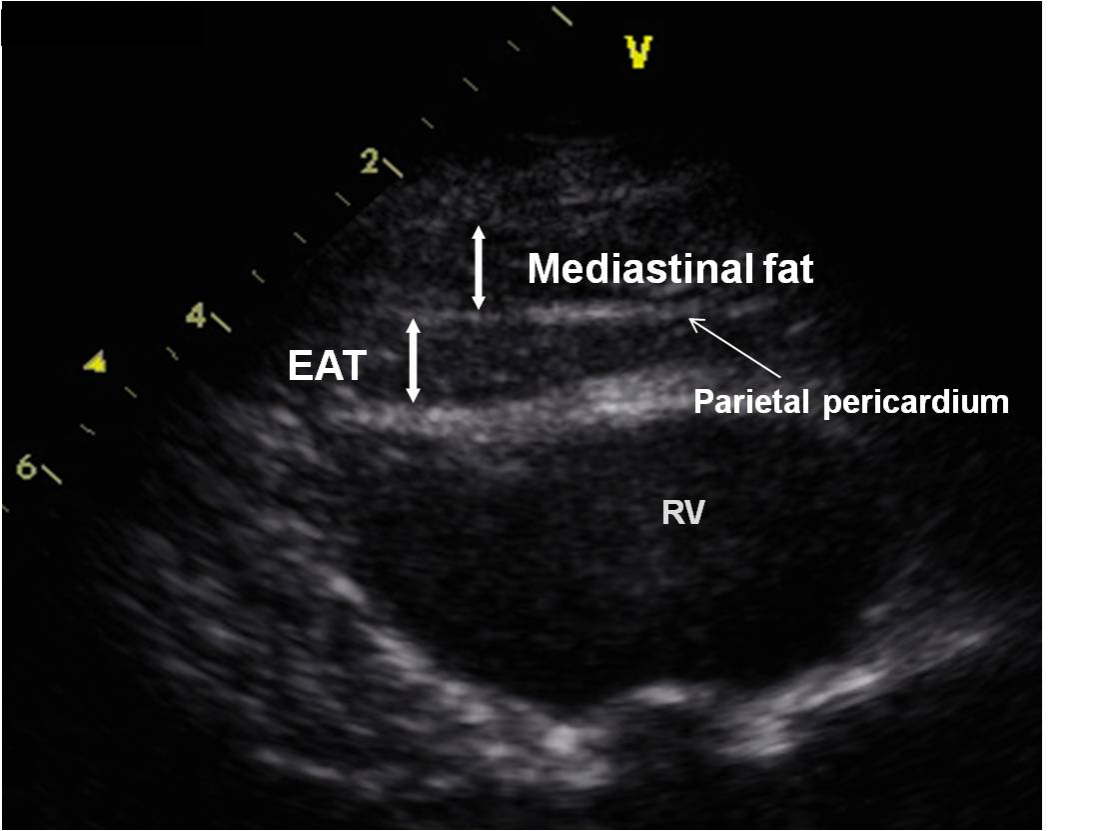
**Echocardiographic measurement of epicardial adipose tissue thickness**. Parasternal long-axis view at the mitral valve level. EAT, epicardial adipose tissue; RV, right ventricle

Two-dimensional transthoracic echocardiography was performed. Recordings of six cycles of the two-dimensional parasternal long-axis were obtained. We enlarged each view for better visualization and accurate measurement of EAT thickness. The measurement of EAT thickness was done offline through the DICOM system and performed on the free wall of the right ventricle (RV) in the still image of a 2-D echocardiogram at end diastole on the parasternal long-axis. We preferred the area of above the RV to measure EAT thickness, because this area is recognized as having the thickest EAT layer. In addition, the parasternal long-and short-axis views allow the most accurate measurement of EAT thickness with optimal cursor beam orientation in each view. We measured the thickest point of EAT in each cycle. The average value of the EAT thickness was calculated (Figure 1).

### Coronary angiography

Quantitative coronary angiographic analysis was performed by one experienced technician who was unaware of the patients' clinical information. Angiographic data were analyzed for the presence of CAD.

### Statistical analysis

The SPSS 12.0 (SPSS inc., Chicago, Illinois, USA) statistical software package was used for all calculations. Data are shown as the mean ± standard deviation (SD) and the median values for continuous variables and as percentages for categorical variables. p < 0.05 indicates statistical significance. We compared the characteristics of patients who had BMI ≥27 kg/m^2 ^(high BMI group) to those with BMI < 27 kg/m^2 ^(non-high BMI group). Comparisons of continuous variables were performed using the unpaired Student t test. As the skewness of the EAT thickness was 0.749 (p < 0.001) using the Kolmogrov-Smirnov test, comparisons of the EAT thickness according to the presence of MS in the high and non-high BMI groups and the presence of CAD in 2 groups were performed using the Wilcoxon rank-sum test rather than the parametric test. Comparisons of the EAT thickness according to the MS score were done using the Kruskal-Wallis test. A receiver operation characteristic (ROC) curve analysis was used to assess the discrimination of MS and CAD based on the EAT thickness according to the BMI. Medcalc 10.4.8 (Medcalc software inc., Mariakerke, Belgium) statistical software was used for comparing the area under curve (AUC) of the ROC curve between groups.

One sample paired t test was performed to evaluate inter- and intraobserver variability in the measurement of EAT from 30 randomly selected subjects.

## Results

### Clinical characteristics

Clinical characteristics according to BMI are summarized in Table 1. Seventy-four percent of patients were included in the non-high BMI group. The non-high BMI group consisted of 478 patients (252 males) with a mean age of 59 ± 11 years. Forty-nine percent of non-high BMI group met the criteria of the MS. The high BMI group was consisted of 165 patients (80 males) with a mean age of 59 ± 11 years. Eighty-one percent of the high BMI group was diagnosed with MS.

**Table 1:** Baseline characteristics according to body mass index (n = 643)

Characteristics	BMI < 27 kg/m^2^ (n = 478)	BMI ≥ 27 kg/m^2^ (n = 165)	p
Male, No	252 (53%)	80 (48%)	NS
Age (year-old)	59 ± 11	59 ± 11	NS
EAT thickness (mm)			
Mean ± SD	3.0 ± 2.4	3.5 ± 2.4	0.01
Median	2.8	3.0	0.01
Body Mass Index (kg/m^2^)	23.7 ± 2.4	29.0 ± 2.1	< 0.001
Waist circumference (cm)			
Male	87.0 ± 8.0	97.1 ± 7.5	< 0.001
Female	84.7 ± 9.1	95.0 ± 7.6	< 0.001
Total cholesterol (mg/dl)	170.6 ± 35.3	182.32 ± 39.2	0.001
LDL cholesterol (mg/dl)	97.0 ± 30.0	104.4 ± 33.7	0.025
Triglyceride (mg/dl)	142.1 ± 83.3	183.1 ± 109.5	< 0.001
HDL cholesterol (mg/dl)			
Male	44.0 ± 10.7	40.3 ± 9.2	0.013
Female	48.3 ± 12.7	47.1 ± 11.3	NS
Fasting glucose (mg/dl)	115.0 ± 33.9	120.0 ± 43.4	NS
Hypertension, No	313 (66%)	125 (76%)	0.012
Diabetes, No	295 (62%)	128 (78%)	< 0.001
Smoking, No	156 (33%)	41 (25%)	0.048
Metabolic Syndrome, No	243 (49%)	133 (81%)	< 0.001
Metabolic Score	2.5 ± 1.3	3.3 ± 1.0	< 0.001
Coronary Artery Disease, No	247 (52%)	74 (45%)	NS
Normal & minimal CAD	230 (48%)	91 (55%)	NS
1 vessel	111 (23%)	31 (19%)	NS
2 vessel	69 (14%)	24 (15%)	NS
3 vessel	65 (14%)	19 (12%)	NS
Medication, No			
Aspirin	115 (24%)	37(22%)	NS
Beta blockers	49 (10%)	19 (12%)	NS
Calcium channel blockers	89 (19%)	33 (20%)	NS
ACE inhibitors	23 (5%)	4 (2%)	NS
AT II receptor blockers	58 (12%)	22 (13%)	NS
Statins	46 (10%)	22 (13%)	NS
Nitrates	51 (11%)	16 (10%)	NS
Diuretics	46 (10%)	18 (11%)	NS

EAT, epicardial adipose tissue; LDL, low-density lipoprotein; HDL, high-density lipoprotein; CAD, coronary artery disease; ACE, angiotensin converting enzyme; AT, angiotensin; SD, standard deviation; NS, not significant

Compared to the non-high BMI group, subjects in the high BMI group had significantly higher rates of hypertension, diabetes and metabolic syndrome and higher levels of total cholesterol, LDL-cholesterol and triglycerides.

The median and mean EAT thickness of 643 patients were 3.0 mm and 3.1 ± 2.4 mm, respectively. In the non-high BMI group, the median and mean EAT thickness were 2.8 mm and 3.0 ± 2.4 mm, respectively. In the high BMI group, the median and mean EAT thickness were 3.0 mm and 3.5 ± 2.4 mm, respectively. The median EAT thickness was significantly increased in the high BMI group compared to that of the non-high BMI group (3.0 vs. 2.8 mm, p = 0.01). The absolute values of the mean paired differences were 0.0200 ± 0.39637, p = 0.773 and 0.0033 ± 0.46866, p = 0.969 for inter- and intraobserver variability of the EAT measurement, indicating good reproducibility.

### Epicardial adipose tissue thickness as a predictor of metabolic syndrome according to body mass index

The median EAT thickness was significantly increased in patients with MS compared to those without MS (3.3 vs. 2.0 mm, p < 0.001). EAT thickness also increased with an increase in the number of MS components (p < 0.001).

**Figure 2:**
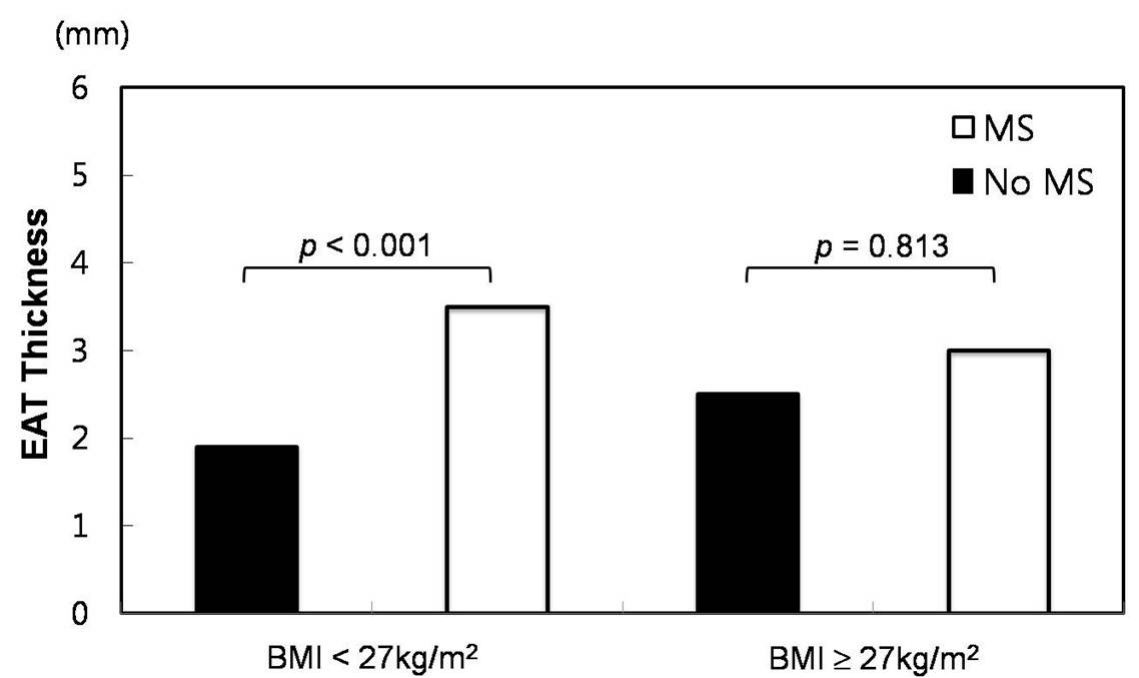
**Distribution of the thickness of epicardial adipose tissue according to body mass index and metabolic syndrome**.

**Figure 3:**
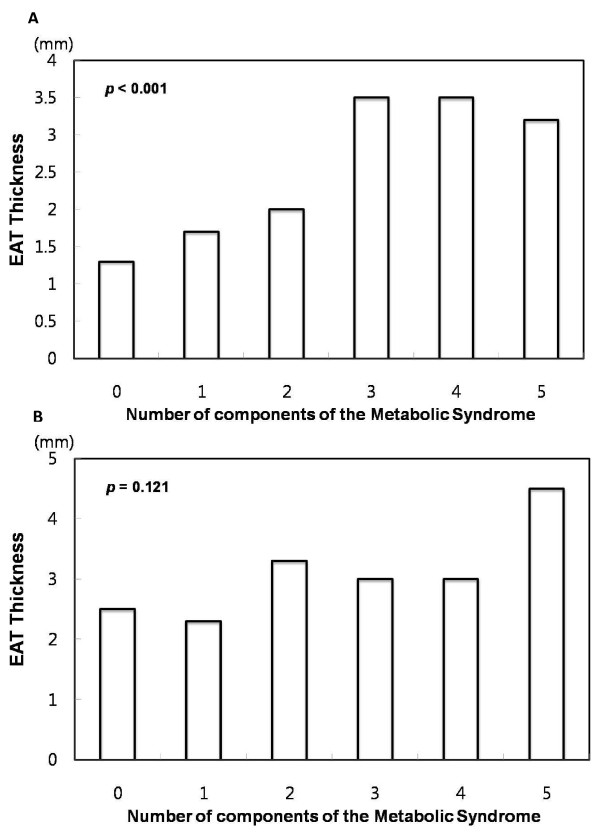
**Distribution of the thickness of epicardial adipose tissue according to body mass index and metabolic score**. (A) Non-high BMI group. (B) High BMI group.

In the non-high BMI group, the median EAT thickness was significantly increased in patients with MS compared to those without MS (3.5 vs. 1.9 mm, p < 0.001, Figure 2). The median EAT thickness values for those with 0, 1, 2, 3, 4, or 5 MS components were 1.3, 1.7, 2.0, 3.5, 3.5, and 3.2 mm, respectively. The median EAT thickness significantly increased with an increase in the number of MS components (p < 0.001, Figure 3-A).

In the high BMI group, the median EAT thickness was not significantly increased in patients with MS compared to those without MS (3.0 vs. 2.5 mm, p = 0.813, Figure 2). The median EAT thickness values for subjects with 0, 1, 2, 3, 4, or 5 MS components were 2.5, 2.3, 3.3, 3.0, 3.0, and 4.5 mm, respectively. The median EAT thickness did not increase with an increase in the number of MS components (p = 0.121, Figure 3-B).

**Figure 4:**
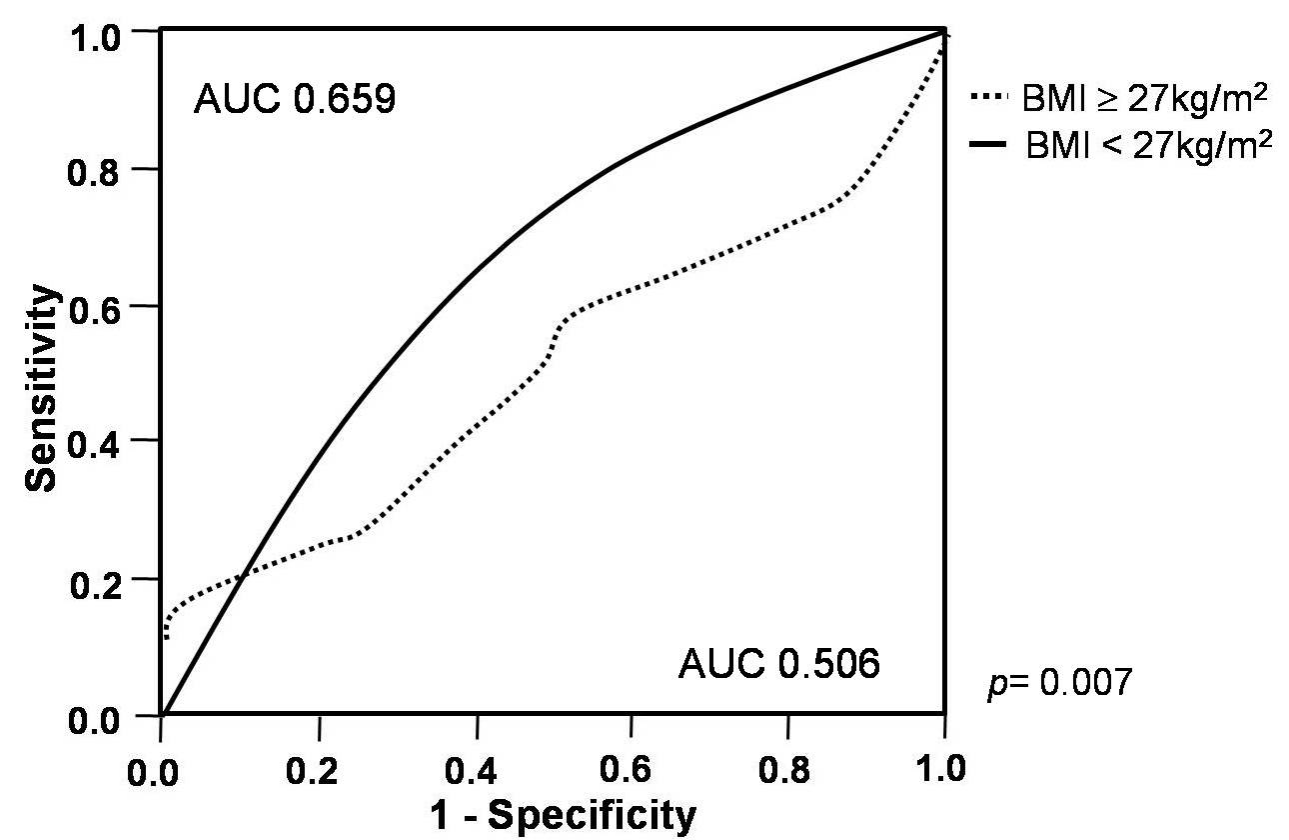
**ROC curve analysis to demonstrate the discriminatory power of the thickness of epicardial adipose tissue in the diagnosis of metabolic syndrome according to body mass index.** AUC, the area under the curve

The ROC curves of the EAT thickness in predicting MS yielded an AUC of 0.659 in the non-high BMI group and 0.506 in the high BMI group. When comparing the AUC of the 2 groups, the AUC to predict MS was significantly higher in the non-high BMI group compared to the high BMI group (p = 0.007, Figure 4).

### Epicardial adipose tissue thickness as a predictor of coronary artery disease according to body mass index

**Figure 5:**
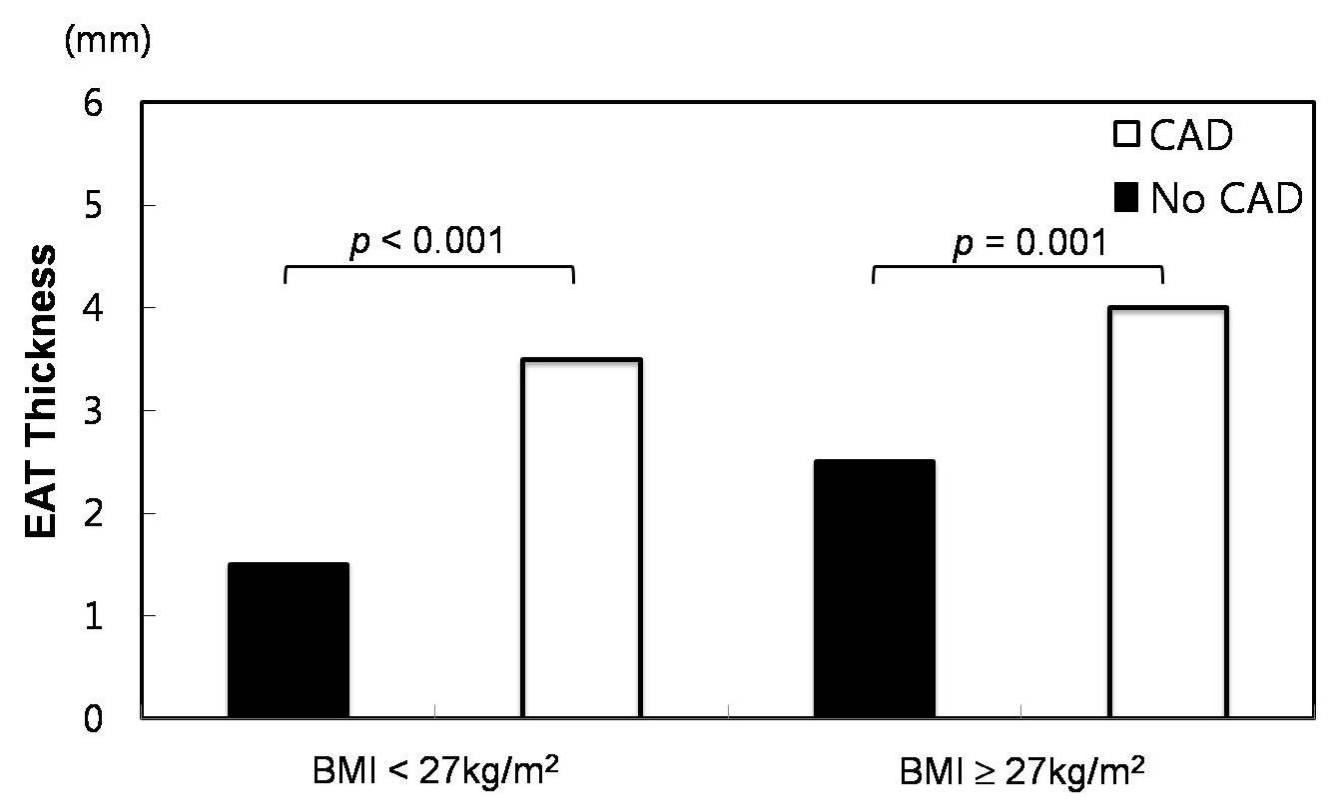
**Distribution of the thickness of epicardial adipose tissue according to body mass index and coronary artery disease**.

**Figure 6:**
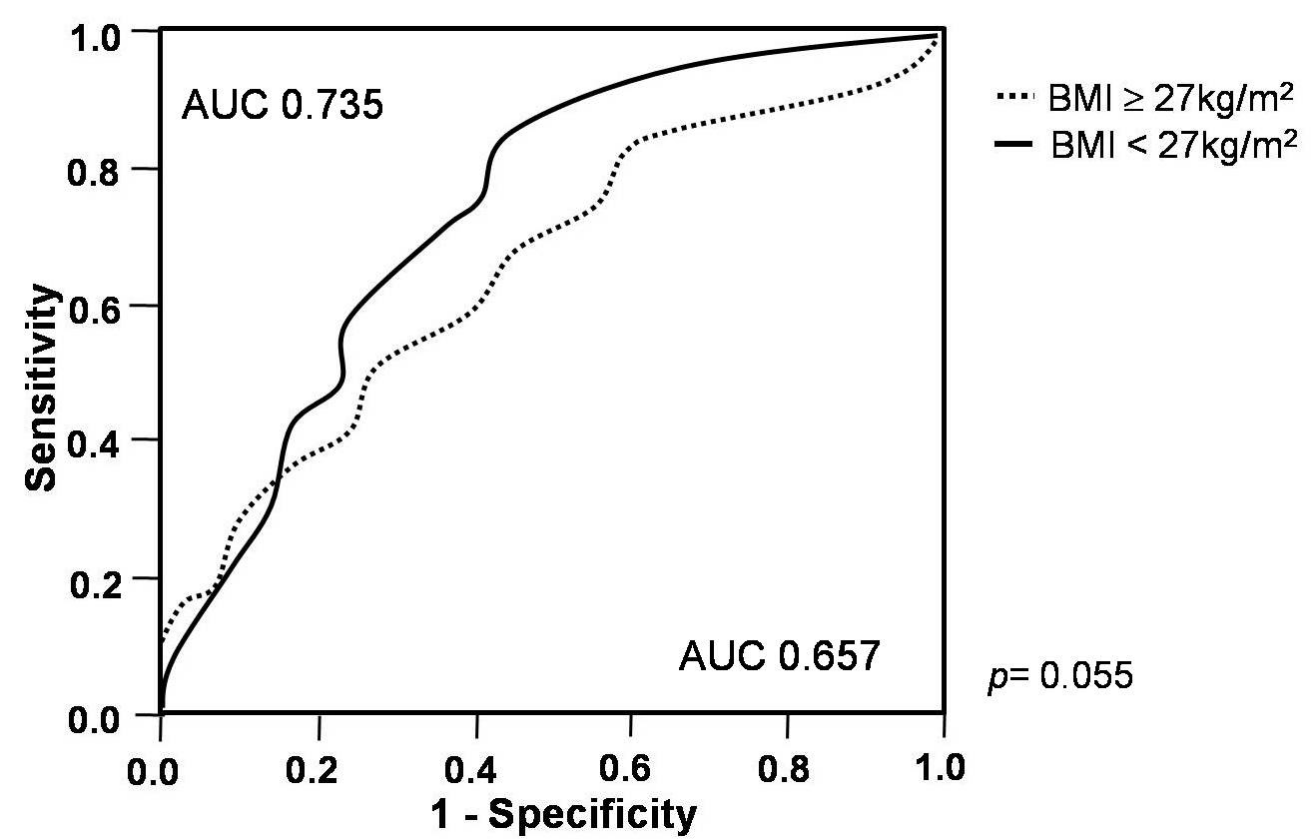
**ROC curve analysis to demonstrate the discriminatory power of the thickness of epicardial adipose tissue in the diagnosis of coronary artery disease according to body mass index.** AUC, the area under the curve

The median EAT thickness was significantly increased in patients with CAD compared to patients without CAD (1.7 vs. 3.5 mm, p < 0.001). When compared to subjects without CAD, patients with CAD in both the non-high and high BMI groups had a median EAT thickness that was significantly increased (3.5 vs. 1.5 mm, p < 0.001 and 4.0 vs. 2.5 mm, p = 0.001, respectively, Figure 5). In addition to well-known CAD risk factors, such as age, smoking, hypertension, and diabetes, EAT thickness was an independent factor associated with CAD in both groups (Table 2). The ROC curves of the EAT thickness in predicting CAD yielded an AUC of 0.657 in the non-high BMI group and 0.735 in the high BMI group. When comparing the AUC of the 2 groups, the ROC curve analysis predicting CAD revealed that the AUC of the BMI < 27 kg/m^2 ^group tended to be larger than that of the BMI ≥27 kg/m^2 ^(p = 0.055, Figure 6).

**Table 2:** Multiple logistic analysis of coronary artery disease risk factors

(A) Non-high BMI group (n = 478)		
**Risk factors**	**Odds ratio (95% CI)**	**p**

Age	2.847 (1.603-5.057)	< 0.001
Male 45 ≥ years		
Female 55 ≥ years		
Smoking	1.576 (1.014-2.448)	0.043
Hypertension	2.115 (1.385-3.230)	< 0.001
Diabetes mellitus	2.139 (1.282-3.569)	0.004
EAT thickness (≥3 mm)	3.504 (2.297-5.346)	< 0.001

**(B) High BMI group (n = 165)**		

**Risk factors**	**Odds ratio (95% CI)**	**p**

Age	6.531 (2.089-20.420)	< 0.001
Male 45 ≥ years		
Female 55 ≥ years		
Smoking	1.775 (0.763-4.129)	0.183
Hypertension	1.276 (0.6-2.712)	0.527
Diabetes mellitus	2.687 (1.222-5.911)	0.014
EAT thickness (≥3 mm)	2.341 (1.136-4.827)	0.021

EAT, epicardial adipose tissue; CI, confidence interval

## Discussion

The present study demonstrated that the power of EAT thickness measured by echocardiography to predict MS was stronger in patients with BMI < 27 kg/m^2^. Our results also demonstrated a strong correlation between EAT thickness and CAD, especially in patients with a non-high BMI. Our study might explain previous inconsistent results from various studies regarding the correlation between EAT and CAD [3,6,7,17]. Recently Gorter et al. reported that, in patients with BMI < 27 kg/m^2^, increased EAT volume and pericoronary fat volume as measured by CT were associated with CAD and extensive coronary artery calcium (CAC) [7]. Although we used a different way to evaluate EAT, using echocardiography to measure EAT thickness, the results were consistent with those of Gorter et al. In addition to these findings, we demonstrated that the predicting value of EAT thickness for MS might be more useful in patients with a non-high BMI. The results of our study suggest that different standards of EAT thickness according to BMI are needed to predict MS and CAD.

In the present study, we used 27 kg/m^2 ^as the cut-off point; any number equal to 27 and above was the BMI for obesity. The relative percentage of body fat at different BMIs clearly varies within populations. The corresponding cut-off point for obesity, based on the assumption that the percentage of body fat in the Asian population is the same as the percentage of body fat in the Western population, was lower than the existing WHO cut-off point for obesity, 30 kg/m^2 ^[18]. Western studies have reported that a BMI ≥ 30 kg/m^2 ^group has a greater risk of death caused by cardiovascular diseases [19,20]. Some recent studies on Asian populations showed that low BMI influences cardiovascular diseases [18,21]. In 2007, the Kangwha Cohort Study reported that the risk of cardiovascular diseases was significantly increased in the BMI ≥ 27 kg/m^2 ^group in the Korean population [22]. This study included the Korean population, so we decided that the cut-off point for obesity was 27 kg/m^2 ^instead of 30 kg/m^2^.

Several biomolecular studies in humans have shown that EAT is metabolically active and an important source of both pro-inflammatory adipokines, such as tumor necrosis factor-α, interleukin 1, interleukin 6 and nerve growth factor, and anti-inflammatory adipokines, such as adiponectin and adrenomedullin [13-15]. The regulation and metabolism of pro- and anti-inflammatory mediators secreted by EAT have been linked to insulin sensitivity [23]. EAT has been shown to express a pathogenic mRNA profile of pro-inflammatory adipokines in patients with CAD [13]. One study showed that expression of adiponectin, an anti-inflammatory adipokine, in EAT was significantly lower in patients with CAD compared to those without CAD [14]. EAT thickness has also been shown to be related to markers of insulin resistance and inflammation [2]. For example, one study demonstrated that obesity leads to adipocyte hypertrophy, which increases the secretion of pro-inflammatory adipokines and decreases the secretion of anti-inflammatory adipokines by EAT [23]. The change of EAT thickness by obesity might have obscured the difference between patients with and without CAD, or with and without MS, in the high BMI group of our study.

The second possible explanation for the weak correlation between the EAT thickness and presence of MS and CAD in the high BMI group, might be the different proportion of the EAT to total amount of VAT according to BMI. Patients with high BMI generally have more VAT, which includes EAT. In the high BMI group, the EAT might make up a smaller proportion of the total VAT compared to the non-high BMI group. In the high BMI group, the EAT thickness by echocardiography might not be representative of the total VAT owing to its smaller proportion of the total VAT. That reason might attenuate the predictive value of EAT thickness by echocardiography for MS and CAD in the high BMI group. Further study is necessary to clarify the different roles of EAT according to BMI. Considering racial and gender differences in addition to BMI, further study will be necessary to set the standards of EAT thickness by echocardiography.

There are several limitations to the present study. First, the study population was highly selective for those who underwent their first coronary angiography due to chest pain. Therefore, the results of the study could not be applied to the general population. Also, it was a cross-sectional study, not a cohort study. In a community-based prospective cohort study, it was reported that pericardial fat predicts incident coronary heart disease independent of conventional risk factors. They evaluated the correlation between pericardial fat and CAD without dividing study subjects according to the degree of obesity [24]. A prospective cohort study might be needed to elucidate the different predicting power of EAT for MS and CAD according to BMI in the general population. Second, EAT thickness by echocardiography does not exactly represent the amount of total EAT. Even though echocardiography is not the optimal method for quantification of EAT, our previous study showed that EAT thickness measured by echocardiography has a good correlation with the total amount of EAT [25]. EAT is true visceral fat deposited around the heart. Epicardial, mesenteric, and omental fat all share the same origin from the splanchnopleurituc mesoderm associated with the gut [26]. EAT thickness measured by echocardiography provides a sensitive and specific measurement of true visceral fat, avoiding the confounding effect caused by subcutaneous fat [27]. As echocardiography is frequently performed in high-risk cardiac patients, EAT thickness measured by echocardiography may be readily available at no extra cost. Therefore, EAT thickness by echocardiography could be applied as an easy and reliable imaging indicator of VAT without radiation exposure [2,3].

## Conclusions

While EAT thickness was significantly increased in patients with MS and CAD regardless of BMI, the power of EAT to predict MS and CAD was stronger in patients with BMI < 27 kg/m^2^. These findings showed that the measurement of EAT thickness by echocardiography might be useful in patients with non-high BMI, especially one less than 27 kg/m^2^.

## Competing interests

The authors declare that they have no competing interests.

## Authors' contributions

All authors participated in the design and coordination of the study, reviewed the analysis and took part in writing the manuscript. They also read and approved the final manuscript.
